# A natural history comparison of *SOD1*-mutant patients with amyotrophic lateral sclerosis between Chinese and German populations

**DOI:** 10.1186/s40035-021-00266-x

**Published:** 2021-10-28

**Authors:** Lu Tang, Johannes Dorst, Lu Chen, Xiaolu Liu, Yan Ma, Kornelia Günther, Sebastian Michels, Kathrin Müller, Axel Freischmidt, Jochen H. Weishaupt, Dongsheng Fan, Albert C. Ludolph

**Affiliations:** 1 Department of Neurology, Peking University Third Hospital, Beijing, 100191 China; 2 Beijing Key Laboratory of Biomarker and Translational Research in Neurodegenerative Diseases, Peking University Third Hospital, Beijing, 100191 China; 3 Department of Neurology, Ulm University, 89081 Ulm, Germany; 4 German Center for Neurodegenerative Diseases (DZNE), Ulm Site, 89081 Ulm, Germany

Currently, there is no effective treatment for amyotrophic lateral sclerosis (ALS), despite the limited efficacy of riluzole [1] and edaravone [2]. *SOD1* (coding for the Cu/Zn superoxide dismutase) is the second most frequent genetic cause for ALS only after *C9orf72* in patients with European ancestry while being the most frequent in Asian ALS populations [3]. Multiple therapeutic approaches have targeted *SOD1*-related ALS, including the antisense oligonucleotide tofersen with promising results in a recent phase I/II trial [4]. Given the clinical heterogeneity among different *SOD1* mutations, in this study, we enrolled genetically confirmed ALS patients with *SOD1* mutations from two prospectively established hospital-based cohorts from China [5] and Germany [6] to clinically characterize distinct *SOD1* mutations and compare related phenotypes between Asians and Caucasians. Because of the explorative nature of this study, all results should be interpreted as hypothesis-generating only rather than confirmatory. No adjustment for multiple testing was made.

We identified 66 Chinese and 84 German ALS patients carrying a total of 69 distinct *SOD1* mutations, including 61 known mutations of *SOD1*, 5 variants of uncertain significance, and 3 likely pathogenic variants. The most frequent mutation in both populations was p.His47Arg (8 Chinese and 2 German). All common mutations featured consistent phenotypes, including an aggressive form of ALS in p.Gly148Asp and slow-progressing forms in p.Glu41Gly, p.His47Arg and p.Asn87Ser (Additional file 1: Table S1). Interestingly, the majority of mutations in the Chinese patients were located in exon 2 while those in the German patients were in exon 4. There was a significant difference in the average age of onset between the Chinese and the German patients carrying mutations in exon 4 (37.4 *vs* 49.9 years, *P* < 0.001). The site of onset, diagnostic delay, and survival did not differ significantly among the exons (Additional file 1: Fig. S1).

Among all patients with *SOD1* mutations, the median (Inter-Quartile Range [IQR]) age of onset was 46.0 (40.0–54.0) years and Chinese patients had younger age of onset (43.0 [38.3–50.0] *vs* 50.0 [41.0–58.0], *P* = 0.002), which is consistent with a previous study [6]. However, the difference was not significant after adjusting for the demographic structures of the identical general ALS populations [6] (*P* = 0.07). The proportion of young-onset ALS, defined as onset between 25 and 45 years, was 47.5% in the two cohorts, and was higher in China (62.5% *vs* 30.7%, *P* < 0.001). The median (IQR) body mass index (BMI) at diagnosis was significantly lower in the Chinese cohort (22.6 [20.9–24.9]) than in the German cohort (25.9 [23.1–28.7], *P* < 0.001); this difference remained significant (*P* = 0.03) after adjusting for the BMI in the identical general ALS populations (median BMI: 23.0 in Chinese and 24.5 in German) [6].

The median (IQR) diagnostic delay was 12.0 (6.0–35.0) months (Chinese: 14.5 [6.0–36.5] *vs* German: 11.0 [6.0–32.0], *P* = 0.59), and the survival was 141.0 (21.0–364.0) months (Chinese: not available due to > 60% censored cases; German: 198.0 [22.0–364.0]; *P* = 0.90). Despite the lower riluzole prescription rate in the Chinese population (28.3% *vs* 81.3%, *P* < 0.001), no survival differences were observed (*P* = 0.90, Table 1).

**Table 1:** Clinical characteristics of Chinese and German ALS patients with *SOD1* mutations

	Total (data available)	China	Germany	*P*	Adjusted *P*^a^
*Nominal variables, n (%)*					
Numbers of subjects	150	66	84		
Sex, male	80 (55.6%) (144)	35 (53.8%)	45 (57.0%)	0.70	
Young-onset ALS (25–45 years)	63 (47.5%) (139)	40 (62.5%)	23 (30.7%)	**< 0.001**	
Site of onset, spinal	116 (94.3%) (123)	57 (91.9%)	59 (96.7%)	0.25	
Pure LMN	16 (20.0%) (80)	9 (17.3%)	7 (25.0%)	0.41	
Riluzole prescription	54 (53.5%) (101)	15 (28.3%)	39 (81.3%)	**< 0.001**	
*Continuous variables, median (IQR)*					
Age of onset (years)	46.0 (40.0–54.0) (139)	43.0 (38.3–50.0)	50.0 (41.0–58.0)	**0.002**	0.07
BMI at diagnosis	23.5 (21.6–26.3) (91)	22.6 (20.9–24.9)	25.9 (23.1–28.7)	**< 0.001**	**0.03**
Diagnostic delay (months)	12.0 (6.0–35.0) (107)	14.5 (6.0–36.5)	11.0 (6.0–32.0)	0.59	
ALSFRS-R at diagnosis	41.0 (35.0–45.0) (116)	42.0 (35.5–46.0)	40.0 (31.0–44.0)	**0.04**	0.70
Early progression rate	0.42 (0.14–0.90) (116)	0.33 (0.15–0.90)	0.46 (0.13–0.93)	0.79	0.29
Late progression rate	0.26 (0.09–0.79) (69)	0.28 (0.08–0.80)	0.17 (0.11–0.77)	0.89	
Survival (months)	141.0 (21.0–364.0) (140)	NA	198.0 (22.0–364.0)	0.90	
Follow-up period	24.0 (7.3–40.8) (88)	30.0 (10.0–42.0)	15.0 (6.0–40.0)	0.06	

Bold *P*-values are significant as *P* < 0.05

LMN, Lower motor neuron

^a^Adjusting for the corresponding data reported in general ALS cohorts of both countries[6]

The majority of cases (94.3%) had spinal onset. There was no difference in the proportion of site of onset between two groups or between males and females. Twenty percent of patients had a pure lower motor neuron phenotype (Chinese: 17.3%, German: 25.0%, *P* = 0.41; Table 1). Some of these patients progressed slowly (p.His47Arg), while others showed an aggressive pattern (p.Ala5Val and p.His44Arg). Male patients had significantly shorter diagnostic delay (*P* = 0.01) and survival (*P* = 0.005) compared to females (Additional file 1: Table S2, Fig. S2).

Because of the significant relationship between diagnostic delay and early and late progression rate (see definition in Additional file 2: Supplementary Methods), only sex, age of onset, site of onset, and late progression rate were included in the multivariate Cox regression analysis, which revealed that patients with bulbar onset (hazard risk 10.31, *P* = 0.01) and higher late progression rate (hazard risk 2.42, *P* = 0.003) had a much shorter survival time (Additional file 1: Table S3, Fig. S3).

The present study has three major implications. First, this study reported for the first time distinct distributions of *SOD1* mutations in Chinese (mainly in exon 2) and German patients (mainly in exon 4), and consistent phenotypes in each of the common mutations. The most common *SOD1* mutations in Germany were p.Arg116Gly (26 patients) and p.Asp91Ala (11), which could be explained by the known founder effects among Caucasian populations, and a similar effect may be involved in Chinese patients carrying p.His47Arg (8 patients). The predominate mutation in North America (p.Ala5Val) [7] was rare in China (only 1 patient), albeit without the same founder haplotype [8], and absent in Germany. Second, the Chinese *SOD1*-mutant patients had a significantly lower age of onset and higher proportion of young-onset cases compared with the German counterparts, which may reflect a higher burden of genetic and environmental risk factors [9]. Third, the known prognostic factors BMI and age of onset for sporadic ALS were not associated with survival in the *SOD1*-carrying patients, indicating the different spectrum of disease-modifying factors.

In the future, it is desirable to establish a detailed genotype–phenotype database of *SOD1* mutation-carriers in different populations in order to clarify the underlying pathomechanisms and to precisely design clinical trials for *SOD1-*related ALS.

## Supplementary Information

**Additional file 1**. **Table S1** Clinical characteristics of common mutations (≥ 2 patients) in Chinese and German ALS patients. **Table S2** Clinical comparison of patients carrying *SOD1* mutation by sex. **Table S3** Cox regression analysis of *SOD1*-mutant patients. **Fig. S1**. Demographic and clinical features of patients carrying *SOD1* mutations by exons. **Fig. S2**. Sex and survival. **Fig. S3**. Prognostic factors and survival.

**Additional file 2**. Supplementary Methods.

## Data Availability

The datasets used and analyzed during the current study are available from the corresponding author on reasonable request.

## Abbreviations

ALS: Amyotrophic lateral sclerosis
BMI: Body mass index
IQR: Interquartile ranges
SOD1: Superoxide dismutase-1

## References

[1] Petrov D, Mansfield C, Moussy A, Hermine O (2017). ALS clinical trials review: 20 years of failure. Are we any closer to registering a new treatment?. Front Aging Neurosci..

[2] Abe K, Aoki M, Tsuji S, Itoyama Y, Sobue G, Togo M (2017). Safety and efficacy of edaravone in well defined patients with amyotrophic lateral sclerosis: a randomised, double-blind, placebo-controlled trial. Lancet Neurol.

[3] Zou Z, Zhou Z, Che C, Liu C, He R, Huang H (2017). Genetic epidemiology of amyotrophic lateral sclerosis: a systematic review and meta-analysis. J Neurol Neurosurg Psychiatry.

[4] Miller T, Cudkowicz M, Shaw PJ, Andersen PM, Atassi N, Bucelli RC (2020). Phase 1–2 trial of antisense oligonucleotide Tofersen for SOD1 ALS. N Engl J Med.

[5] Tang L, Ma Y, Liu X, Chen L, Fan D (2019). Better survival in female SOD1-mutant patients with ALS: a study of SOD1-related natural history. Transl Neurodegener.

[6] Dorst J, Chen L, Rosenbohm A, Dreyhaupt J, Hübers A, Schuster J (2019). Prognostic factors in ALS: a comparison between Germany and China. J Neurol.

[7] Bali T, Self W, Liu J, Siddique T, Wang LH, Bird TD (2017). Defining SOD1 ALS natural history to guide therapeutic clinical trial design. J Neurol Neurosurg Psychiatry.

[8] Tang L, Ma Y, Liu X, Chen L, Fan D (2018). Identification of an A4V SOD1 mutation in a Chinese patient with amyotrophic lateral sclerosis without the A4V founder effect common in North America. Amyotroph Lateral Scler Frontotemporal Degener.

[9] Sabatelli M, Madia F, Conte A, Luigetti M, Zollino M, Mancuso I (2008). Natural history of young-adult amyotrophic lateral sclerosis. Neurology.

