# Supplementary material for: A natural history comparison of SOD1-mutant patients with amyotrophic lateral sclerosis between Chinese and German populations
Source: Transl Neurodegener. 2021 Oct 28;10:42. doi: 10.1186/s40035-021-00266-x (PMC8555265; doi:10.1186/s40035-021-00266-x)
Supplement: Supplementary file 1 — Additional file 1. Table S1 Clinical characteristics of common mutations (≥ 2 patients) in Chinese and German ALS patients. Table S2 Clinical comparison of patients carrying SOD1 mutation by sex. Table S3 Cox regression analysis of SOD1-mutant patients. Fig. S1. Demographic and clinical features of patients carrying SOD1 mutations by exons. Fig. S2. Sex and survival. Fig. S3. Prognostic factors and survival. [file 40035_2021_266_MOESM1_ESM.docx]

**Table S1** Clinical characteristics of common mutations (≥ 2 patients) in Chinese and German ALS patients

|  | **Number of Patients** | | **Age of Onset**  **(years, mean ± SD)** | | **Survival**  **(months, mean ± SD)** | |
| --- | --- | --- | --- | --- | --- | --- |
|  | **Chinese** | **German** | **Chinese** | **German** | **Chinese** | **German** |
| ***Found in both populations*** | | | | | | |
| p.Glu41Gly | 1 | 1 | 36 | 41 | 71* | 162 * |
| p.His44Arg | 3 | 2 | 40.7 ± 2.9 | 58 * | 38.3 ± 43.3 * | 9 * |
| p.His47Arg | 8 | 2 | 50.8 ± 10.1 | 45 ±14.1 | 108 ± 55.5 * | 210 ± 12.7 * |
| p.Leu85Phe | 2 | 1 | 38 ± 0 | 33 | 42 ± 2.8 | 14 |
| p.Asn87Ser | 1 | 1 | 51 | 46 | 82 * | 100 * |
| p.Ile114Thr | 1 | 1 | 38 | 64 | 18 * | 63 * |
| p.Gly148Asp | 1 | 1 | 43 | 58 | 10 | 7 |
| ***Found only in Chinese patients*** | | | | | | |
| p.Val15Met | 2 |  | 54.5 ± 7.8 |  | 59.0 ± 14.1 * |  |
| p.Gly17Ala | 2 |  | 35 ± 8.5 |  | 106 ± 24.0 * |  |
| p.Gly42Asp | 3 |  | 46.7 ± 3.8 |  | 67 ± 34.0 * |  |
| p.Gly42Ser | 3 |  | 43.3 ± 11.5 |  | 15.7 ± 7.1 * |  |
| p.Val48Ala | 2 |  | 51.5 ± 3.5 |  | 29 ± 24.0 * |  |
| p.Leu107Phe | 3 |  | 39.7 ± 5.7 |  | 58.5 ± 55.9 * |  |
| p.Cys112Tyr | 2 |  | 27.5 ± 10.6 |  | 88 ± 94.8 * |  |
| p.Gly142Ala | 3 |  | 48.3 ± 5.7 |  | 34.3 ± 8.0 * |  |
| p.Ile150Val | 2 |  | 42 ± 0 |  | 95.5 ± 84.1 * |  |
| ***Found only in German patients*** | | | | | | |
| p.His49Arg |  | 2 |  | 48 ± 7.1 |  | 203.5 ± 227.0 |
| p.Gly73Ser |  | 3 |  | 45.5 ± 26.2 |  | 45 ± 48.1 * |
| p.Val88Ala |  | 2 |  | 61 ± 0 |  | 35.5 ± 2.8 |
| p.Asp91Ala |  | 11 |  | 48.9 ± 16.0 |  | 107.9 ± 62.6 * |
| p.Glu101Lys |  | 5 |  | 37 ± 8.2 |  | 188.2 ± 151.6 * |
| p.Ile105Phe |  | 2 |  | 42.5 ± 20.5 |  | 264.5 ± 20.5 * |
| p.Ile113Thr |  | 2 |  | 65 ± 7.1 |  | 15 ± 8.5 |
| p.Arg116Gly |  | 26 |  | 52.4 ± 10.5 |  | 28.0 ± 21.2 * |
| p.Leu145Phe |  | 6 |  | 51.3 ± 6.4 |  | 53.8 ± 30.0 * |
| p.Val149Gly |  | 2 |  | 51.5 ± 3.5 |  | 10 ± 2.8 |

*includes censored data (at least 1 patient still alive or missing data); SD, standard deviatio

**Table S2** Clinical comparison of patients carrying SOD1 mutation by sex

|  | **Male** | **Female** | ***P*** |
| --- | --- | --- | --- |
| ***Nominal variables, n (%)*** | | | |
| Numbers of subjects | 80 | 64 |  |
| Sex, male | - | - | - |
| Young-onset ALS  (25–45 years) | 30 (39.5%) | 33 (52.4%) | 0.10 |
| Site of onset, spinal | 66 (94.3%) | 50 (94.3%) | 0.99 |
| Pure LMN | 8 (20.0%) | 8 (20.0%) | 1.00 |
| Riluzole prescription | 32 (60.4%) | 22 (45.8%) | 0.14 |
| ***Continuous variables, median (IQR)*** | | | |
| Age of onset (years) | 49.5 (41.0-55.8) | 43.0 (39.0-51.0) | 0.08 |
| BMI at diagnosis | 24.2 (21.7-26.2) | 22.9 (21.4-27.3) | 0.49 |
| Diagnostic delay (months) | 11.0 (5.0-24.8) | 15.0 (8.0-47.0) | **0.01** |
| ALSFRS-R at diagnosis | 41.0 (35.0-45.0) | 40.0 (34.0-44.0) | 0.35 |
| Early progression rate  (onset to first visit) | 0.49 (0.20-0.82) | 0.31 (0.13-1.00) | 0.45 |
| Late progression rate  (first to last visit) | 0.30 (0.11-0.90) | 0.20 (0.09-0.53) | 0.32 |
| Survival (months) | 60.0 (17.0-250.0) | 248.0 (44.0-419.0) | **0.005** |
| Follow-up period | 13.0 (7.0-31.0) | 30.0 (9.0-45.0) | 0.12 |

**Table S3** Cox regression analysis of *SOD1*-mutant patients

|  | **Univariate** | | | **Multivariate** | | |
| --- | --- | --- | --- | --- | --- | --- |
| **Variable** | **HR (95%CI)** | ***P* Value** | **HR (95%CI)** | | ***P* Value** |  |
| Ethnicity |  |  |  | |  |  |
| Chinese | 1.00 |  |  | |  |  |
| German | 1.04 (0.61-1.75) | 0.90 | - | | - |  |
| Sex |  |  |  | |  |  |
| Female | 1.00 |  |  | |  |  |
| Male | 2.10 (1.23-3.57) | **0.006** | 1.77 (0.60-5.21) | | 0.30 |  |
| Age of onset (years) | 1.02 (1.00-1.04) | 0.054 | 1.01 (0.97-1.06) | | 0.68 |  |
| Site of onset |  |  |  | |  |  |
| Spinal | 1.00 |  |  | |  |  |
| Bulbar | 3.74 (1.58-8.86) | **0.003** | 10.31 (1.70-62.54) | | **0.01** |  |
| Diagnostic delay (months) | 0.93 (0.90-0.96) | **<0.001** | - | |  |  |
| BMI (kg/m^2^) | 0.96 (0.87-1.05) | 0.34 | - | |  |  |
| Early progression rate (point/month) | 1.15 (1.04-1.27) | **0.009** | - | |  |  |
| Late progression rate (point/month) | 2.77 (1.64-4.67) | **<0.001** | 2.42 (1.36-4.28) | | **0.003** |  |
| riluzole | 1.00 (0.52-1.90) | 1.00 | - | | - |  |


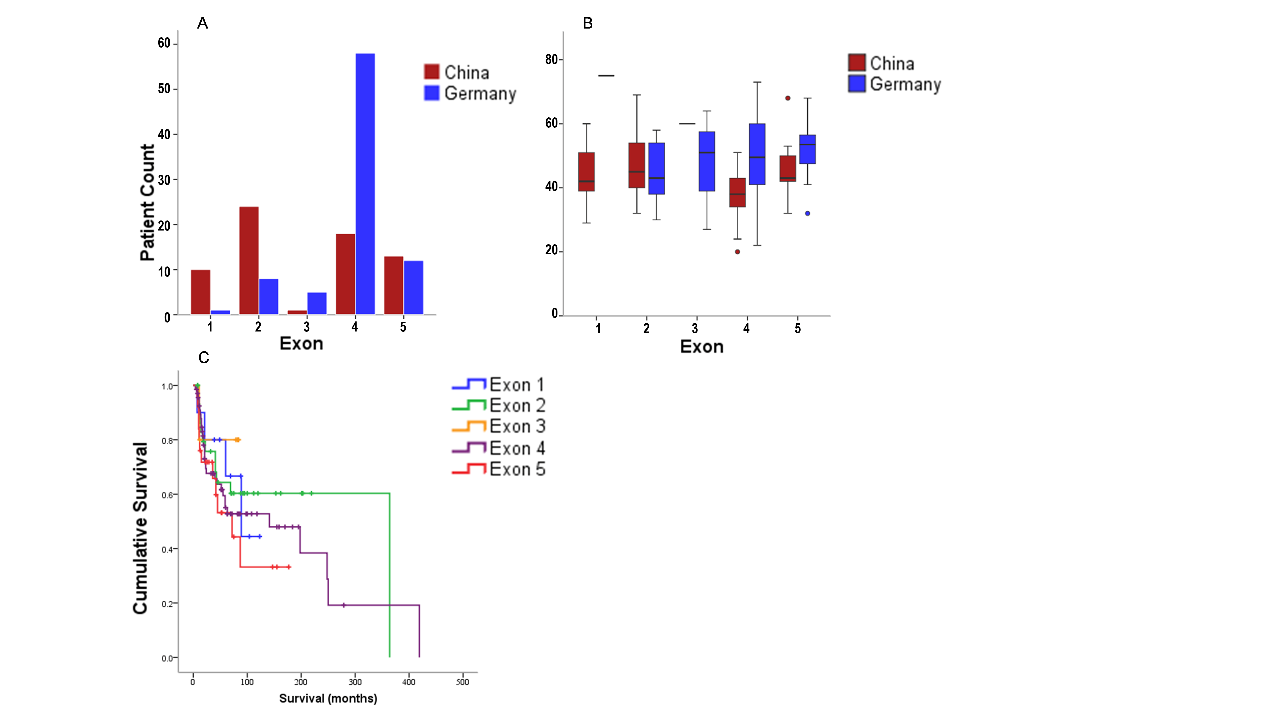


***Figure S1.*** Demographic and clinical features of patients carrying *SOD1* mutations by exons. (A) Exonal distribution of patients with *SOD1* mutations in China and Germany. The distribution was significantly different (*P* <0.001). (B) Age of onset of Chinese and German patients per exon, revealing a significant difference (*) in exon 4 (China: mean 37.4 years; Germany: 49.9 years, *P* <0.001). (C) Survival curves per exon showed no significant differences (*P* = 0.64).


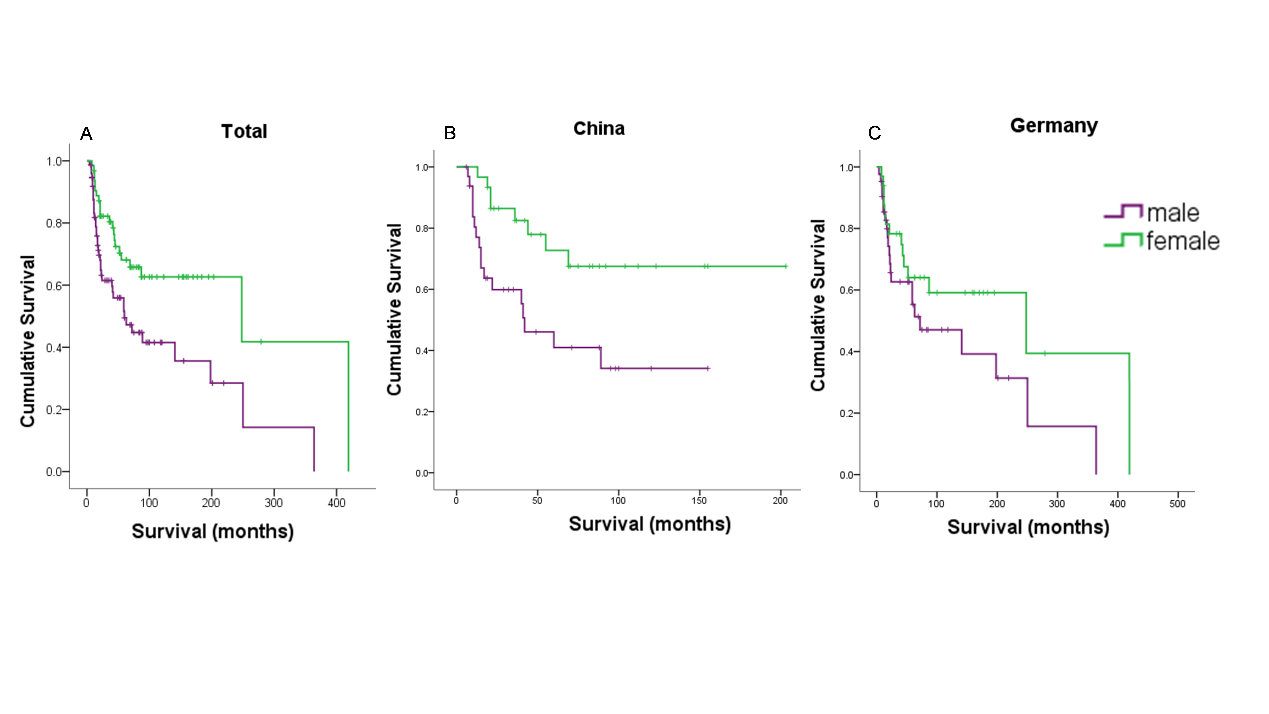


***Supplementary Figure S2.*** Sex and survival. Kaplan Meier curves show survival for male (purple) and female (green) patients overall (A: *P* = 0.005), in China (B: *P* = 0.009), and Germany (C: *P* = 0.15).


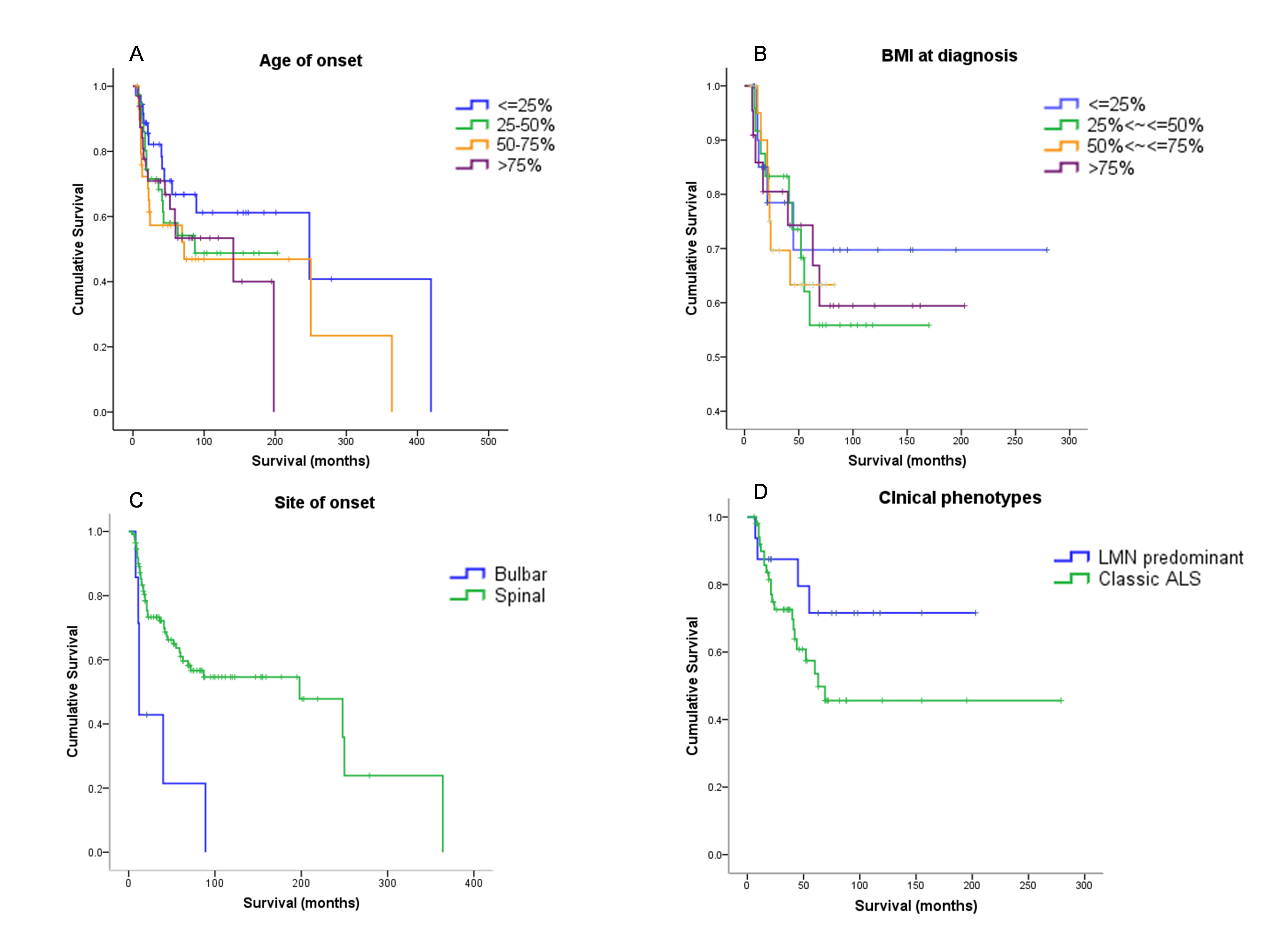


***Figure S3.*** Prognostic factors and survival. Kaplan Meier curves show the effect of age of onset (A, *P* = 0.33), BMI at diagnosis (B, *P* = 0.97), site of onset (C, *P* = 0.001), and clinical phenotype (D, *P* = 0.15) on survival. LMN = lower motor neuron.
