# Supplementary material for: A natural history comparison of SOD1-mutant patients with amyotrophic lateral sclerosis between Chinese and German populations
Source: Transl Neurodegener. 2021 Oct 28;10:42. doi: 10.1186/s40035-021-00266-x (PMC8555265; doi:10.1186/s40035-021-00266-x)
Supplement: Supplementary file 2 — Additional file 2. Supplementary Methods. [file 40035_2021_266_MOESM2_ESM.docx]

***Supplementary Methods***

**Participants Inclusion and Variables of interest**

Chinese patients were recruited at a national referral motor neuron disease (MND) center at Department of Neurology, Peking University Third Hospital (PUTH), Beijing between 2007 to 2013, which has been described previously[1]. Roughly about one fifth of the patients in this center were recruited from Beijing metropolitan area[2]. German patients were collected from the database of the German network for MND between 1999 and 2019. The German MND network is a consortium consisting of 21 centers specialized in ALS. The ALS center of PUTH and the German ALS network have established a long-lasting cooperation over the last years, which included the synchronization of databases in order to obtain comparable data [3].

Written informed consent was obtained from all patients for participation in clinical and genetic studies, which were approved by the respective local institutional ethics committees. Patients were diagnosed with definite, probable, or possible ALS according to Airlie House diagnostic criteria [4] and had a known *SOD1* mutation, a SOD1 variant of uncertain significance, or a likely pathogenic SOD1 variant according to American College of Medical Genetics (ACMG) Standards and Guidelines [5]. Patients were followed up every 3-6 months (Germany: visits in outpatient clinics; China: phone call follow-ups).

Demographic information included sex, date of birth, month/year of disease onset, date of diagnosis, date of last follow-up, month/year of death or invasive ventilation (if applicable, both were defined as endpoint events), and self-reported ethnicity and family history of ALS. Clinical information included site of onset, first clinical symptom, predominant affection of UMN or LMN, body mass index (BMI) at diagnosis, and ALS-Functional Rating Scale Revised (ALSFRS-R) [6] at each visit/follow-up.

Diagnostic delay was defined as the interval between onset (first paresis) and diagnosis. Disease progression rate was defined as loss of ALSFRS-R score per month between diagnosis and first visit/follow-up (early progression rate) as well as between first and last visit/follow-up (late progression rate). Survival was defined as the time between onset and endpoint events.

**DNA sequencing and analysis**

DNA was extracted from blood samples collected from outpatient visits or in hospital, usually at first visit. For Chinese cases, both apparently sporadic patients and the probands of families were consecutively included in genetic analysis. Sequence analysis has been previously partly published[1]. Briefly, Sanger sequencing was performed for all coding exons and flanking 50bps of *SOD1* (NM 000454.5). In Germany, patients with a positive family history of ALS and patients with unusual features (such as young age of onset) were routinely tested. Additionally, sporadic patients without these features who wished genetic testing via the German MND network were tested as well. The patients were screened by Sanger sequencing for all coding exons of *SOD1*(NM 000454.5). The novel variants identified in the present study were evaluated according to the ACMG Standards and Guidelines.

**Statistical analysis**

Descriptive statistics (frequencies and percentages, mean and 95% confidence intervals [95% CI], or medians and interquartile ranges [IQRs], as appropriate) were used to characterize the study participants. The Chi-square test was used for nominal variables. Independent-sample student’s t-test or ANOVA test was applied for analyzing normally distributed continuous variables, and non-parametric Mann-Whitney U test or Kruskal-Wallis test for non-normally distributed variables. Kaplan-Meier curves and log-rank test were applied to determine the effect of demographic or clinical parameters on survival. A Cox multivariate proportional hazards model was applied to account for prognostic variables. Statistical significance was set at p<0.05 (two-sided). Because of the explorative nature of this study, the results from the statistical analysis have to be interpreted as hypothesis generating only but not as confirmatory. No adjustment for multiple testing was applied.

**References**

1. Tang L, Ma Y, Liu X, Chen L, Fan D. Better survival in female SOD1-mutant patients with ALS: a study of SOD1-related natural history. Transl Neurodegener. 2019; 8(1).

2. Chen L, Zhang B, Chen R, Tang L, Liu R, Yang Y, Yang Y, Liu X, Ye S, Zhan S*, et al*. Natural history and clinical features of sporadic amyotrophic lateral sclerosis in China. Journal of Neurology, Neurosurgery & Psychiatry. 2015; 86(10):1075-81.

3. Dorst J, Chen L, Rosenbohm A, Dreyhaupt J, Hübers A, Schuster J, Weishaupt JH, Kassubek J, Gess B, Meyer T*, et al*. Prognostic factors in ALS: a comparison between Germany and China. J Neurol. 2019; 266(6):1516-25.

4. Brooks B, Miller R, Swash M, Munsat T, Diseases. WFON. El Escorial revisited: revised criteria for the diagnosis of amyotrophic lateral sclerosis. Amyotroph Lateral Scler Other Motor Neuron Disord. 2000; 1:293-9.

5. Richards S, Aziz N, Bale S, Bick D, Das S, Gastier-Foster J, Grody WW, Hegde M, Lyon E, Spector E*, et al*. Standards and guidelines for the interpretation of sequence variants: a joint consensus recommendation of the American College of Medical Genetics and Genomics and the Association for Molecular Pathology. Genet Med. 2015; 17(5):405-24.

6. Cedarbaum JM, Stambler N, Malta E, Fuller C, Hilt D, Thurmond B, Nakanishi A. The ALSFRS-R: a revised ALS functional rating scale that incorporates assessments of respiratory function. BDNF ALS Study Group (Phase III). J Neurol Sci. 1999; 169:13-21.
